# A New Theory of Animal Heat

**Published:** 1854-11

**Authors:** 


From the Lancet]

*A New Theory of Animal Heat.*

At a late meeting of the Physiylogical Society of London, Dr.

Winn read a paper on "The Elasticity of Arteries considered as a Cause of Animal Heat," and presented a series of experiments made by him with a view of proving the theory.

He states that about seventeen years ago, whilst experimenting with the caoutchouc, he was struck with the property which that substance possessed of evolving heat when suddenly elongated, and was led to infer the probability of other bodies being similarly endowed. The elastic coat of arteries, especially, appeared to be one of the substances likely to exhibit the califactory principle; and in the event of this being the case, it would not be unreasonable to infer that the incessant contractions and dilatations of the arteries during life must form an efficient cause of animal heat.

The following experiments, made with portions of the aorta of a bullock, somewhat verify the conjecture, and are, at any rate, worthy of notice:

Having cut off a circular portion of the descending aorta, an inch in length, he laid it open, and removed its external and internal covering. He then pulled it to and fro, with a continuous jerking motion, (in imitation of the cystole and diastole of the heart,) for one minute. He then placed it in the bulb of a thermometer, and found that the mercury had risen *two degrees*. To be certain that the increased heat did not arise from any source, he took the precaution of wrapping his finger with a double fold of flannel, in order to prevent a communication of heat from the body. He also covered his mouth with a handkerchief, to guard against the warm breath affecting the experiment. There was no fite in the room, the temperature being 36°.

The principal difficulty in this investigation appears to be the moisture of the artery, which, by its evaporation, had a tendency to carry off a portion of the heat. By carefully drying it with a cloth, however, this impediment was partially removed.

His attention was often arrested during the experiment by other mechanical analogies between the elastic coat of arteries and caoutchouc. They both would bear elongation to twice their length, and would return to their usual dimensions with considerable force and the same snapping sound.

From the preceding observations, Dr. Winn concluded that the generation of animal heat could be satisfactorily explained. Physiologists had proved that a certain portion of animal heat was the result of chemical changes in the blood. Yet they confessed that a residuum of heat could not be thus accounted for, and Dr. Winn proposes to refer this residuum to the mechanical action of the arteries. Although difficult to measure the exact amount given during each arterial beat: yet, if the development of only a small quantity was admitted, it necessarily followed, from the incessant action of the arteries, that the body, unless cooled by the functions of the skin and lungs, would soon become preternaturally hot.

Dr. Winn presents the following physiological and pathological facts as corroborative of this theory:

1st. The minute distribution of the arteries to every part of the system, thus ensuring an equal distribution of heat. 2d. The *rigidity* of the arterial system, in old age, as being the probable cause of the diminution of animal heat. 3d. The increased warmth of the body after exercise seemed to be explicable upon the principle of the increased force of the arteries. 4th. When there existed any arterial excitement, and especially if the functions of the lungs (or skin) were at fault, the heat of the body would be greatly increased. 5th. Medicines which reduced the force of the heart and arteries diminished animal heat. 6th. The heat of local inflammations, when there was no constitutional disturbance, could only be accounted for in this way, as the arteries in the neighbourhood of the disease would be throbbing violently, whereas the capillaries, which are supposed to play so important a part in the chemical theory, are generally considered to have their action impeded.

